# Assessing a Sleep Interviewing Chatbot to Improve Subjective and Objective Sleep: Protocol for an Observational Feasibility Study

**DOI:** 10.2196/45752

**Published:** 2023-05-11

**Authors:** Ting Su, Rafael A Calvo, Melanie Jouaiti, Sarah Daniels, Pippa Kirby, Derk-Jan Dijk, Ciro della Monica, Ravi Vaidyanathan

**Affiliations:** 1 Department of Mechanical Engineering Imperial College London London United Kingdom; 2 Dyson School of Design Engineering Imperial College London London United Kingdom; 3 Care Research & Technology Centre UK Dementia Research Institute London United Kingdom; 4 Department of Brain Sciences Imperial College London London United Kingdom; 5 Department of Therapies Imperial College Healthcare NHS Trust London United Kingdom; 6 Surrey Sleep Research Centre School of Biosciences University of Surrey Guildford United Kingdom

**Keywords:** automated chatbot, behavior analysis, conversational agents, older adults, sleep disorders, sleep interview

## Abstract

**Background:**

Sleep disorders are common among the aging population and people with neurodegenerative diseases. Sleep disorders have a strong bidirectional relationship with neurodegenerative diseases, where they accelerate and worsen one another. Although one-to-one individual cognitive behavioral interventions (conducted in-person or on the internet) have shown promise for significant improvements in sleep efficiency among adults, many may experience difficulties accessing interventions with sleep specialists, psychiatrists, or psychologists. Therefore, delivering sleep intervention through an automated chatbot platform may be an effective strategy to increase the accessibility and reach of sleep disorder intervention among the aging population and people with neurodegenerative diseases.

**Objective:**

This work aims to (1) determine the feasibility and usability of an automated chatbot (named MotivSleep) that conducts sleep interviews to encourage the aging population to report behaviors that may affect their sleep, followed by providing personalized recommendations for better sleep based on participants’ self-reported behaviors; (2) assess the self-reported sleep assessment changes before, during, and after using our automated sleep disturbance intervention chatbot; (3) assess the changes in objective sleep assessment recorded by a sleep tracking device before, during, and after using the automated chatbot MotivSleep.

**Methods:**

We will recruit 30 older adult participants from West London for this pilot study. Each participant will have a sleep analyzer installed under their mattress. This contactless sleep monitoring device passively records movements, heart rate, and breathing rate while participants are in bed. In addition, each participant will use our proposed chatbot MotivSleep, accessible on WhatsApp, to describe their sleep and behaviors related to their sleep and receive personalized recommendations for better sleep tailored to their specific reasons for disrupted sleep. We will analyze questionnaire responses before and after the study to assess their perception of our proposed chatbot; questionnaire responses before, during, and after the study to assess their subjective sleep quality changes; and sleep parameters recorded by the sleep analyzer throughout the study to assess their objective sleep quality changes.

**Results:**

Recruitment will begin in May 2023 through UK Dementia Research Institute Care Research and Technology Centre organized community outreach. Data collection will run from May 2023 until December 2023. We hypothesize that participants will perceive our proposed chatbot as intelligent and trustworthy; we also hypothesize that our proposed chatbot can help improve participants’ subjective and objective sleep assessment throughout the study.

**Conclusions:**

The MotivSleep automated chatbot has the potential to provide additional care to older adults who wish to improve their sleep in more accessible and less costly ways than conventional face-to-face therapy.

**International Registered Report Identifier (IRRID):**

PRR1-10.2196/45752

## Introduction

### Overview

Evidence exists for a bidirectional relationship between sleep disorders in the aging population and people with neurodegenerative disease [1-3]. Specifically, between 40% and 70% of the older population report difficulties in sleep onset or maintenance and insomnia symptoms [4-6], which increase their risk of developing neurodegenerative disorders. Similarly, there is a high prevalence of sleep disorders, such as sleep apnea and circadian rhythm disorders, among people with neurodegenerative disorders [7-10], where sleep disorders can accelerate cognitive decline [11,12]. Moreover, neurodegenerative disorders affect not only patients but their entire households, especially the primary carer. Indeed, studies [13-16] have shown that approximately two-thirds of older adult caregivers have some form of sleep disturbance. Thus, early intervention of sleep disorders among older adults can improve their quality of life, reduce their risk of developing neurodegenerative disorders, and prevent further complications among household members.

Researchers have conducted clinical trials using cognitive behavioral therapy [17-19] and motivational interviews [20-22] to improve sleep quality among older people, people with neurodegenerative disorders, and their carers. However, one-to-one individual therapies (in-person or remote) and face-to-face group therapies may be costly and difficult to access.

Several digital insomnia tracking and treatment systems [23,24] have been developed and evaluated. For example, Zaslavsky et al [22] developed a mobile health (mHealth) monitoring and self-management app. mHealth monitors sleep quality through the wearable Fitbit Charge 2 and automatically sends text messages that provide weekly information and motivational feedback. Zaslavsky et al [22] showed that their proposed mobile app mHealth, combined with motivational interviews conducted by telephone, can significantly improve participants’ self-reported sleep quality after 19 weeks of participation. In addition, Aji et al [23] showed that a self-reporting mobile app (SleepFix), connected to a wearable sleep-tracking device, can encourage users’ engagement with the app’s self-reporting functionality in a pilot randomized controlled trial.

However, these studies only combine digital sleep quality monitoring with a digital self-reporting mechanism or periodic text messages to encourage users to engage in sleep quality reporting, good sleep habits, and routine. They do not engage in conversations that may further encourage information sharing and behavioral changes. To engage more actively with users, Rick et al [24] proposed a Telegram-based chatbot, SleepBot, that dynamically enquires about users’ sleep information based on preprogrammed questions. Aarts et al [25] proposed a chatbot Snoozy for children aged 8-12 years with sleep difficulties, which asks children how their sleep was, the reasons for it if they slept poorly, and what behavioral methods they have tried to help with their sleep. Aarts et al [25] showed that a chatbot could help collect subjective reports from children over sustained period instead of traditional diaries.

In this feasibility study, we propose a sleep intervention chatbot design named MotivSleep. Our proposed MotivSleep first triages participants’ sleep based on information gathered from a contactless sleep-tracking analyzer. Then, MotivSleep starts conducting sleep interviews only when the sleep analyzer identifies a bad night’s sleep. The automatic sleep interview asks participants about any potential adverse factors that might have affected their sleep the night before, prior to using this information to provide personalized recommendations for better sleep (ie, healthy habits that you can practice during the day to help you get a good night’s sleep) [26] and encouraging them to maintain good sleep habits.

### Design Engineering Process

This work is built upon automated and manual monitoring and care procedures followed by the Care Research and Technology Center (CRT), part of the UK Dementia Research Institute (UK DRI). The design of our proposed MotivSleep chatbot involved an advisory group with patients and health care professionals from its conception, following the identified key characteristics in developing human-centered care technologies [27]. Specifically, carers, health professionals, occupational therapists, sleep specialists, chatbot researchers, and designers have come together in 2 workshops where the needs and values of the stakeholders were discussed [28]. We also requested input from the advisory board, conducted interviews with people living with dementia and their carers, conducted patient and public involvement and engagement sessions with healthy older adults, and surveyed results to develop initial dialogue plans that can conduct sleep interviews through chatbot.

Based on information and advice from the groups mentioned above, we built a sleep management chatbot integrated with WhatsApp. This internationally available messaging platform provides instant text and voice messaging services through the American company Meta Platforms. To access the proposed MotivSleep chatbot, we will gather the participants’ phone numbers and add them to the MotivSleep database, where messages will be sent out automatically at the user’s preferred time, potentially daily. Additionally, we will use the Withings sleep analyzer, a commercially available contactless device, to monitor and track participants’ objective sleep parameters. The sleep analyzer can be installed under the participant’s mattress, and the information gathered can be accessed through an API. The Withings sleep analyzer is chosen due to its passive recording ability (ie, it does not need recharging or adjustments once installed) and previous collaboration between UK DRI and Withings that provides easy integration. Additionally, the Withings sleep analyzer has been evaluated and showed comparable results to actigraphy and polysomnography based sleep assessment, in older people [29]. In this study, we will analyze participants’ sleep data to monitor the participants’ objective sleep parameters based on respiration, heart rate, snoring, and apnea episodes. This analysis triggers the conversation stage of our proposed MotivSleep chatbot.

**Figure 1:**
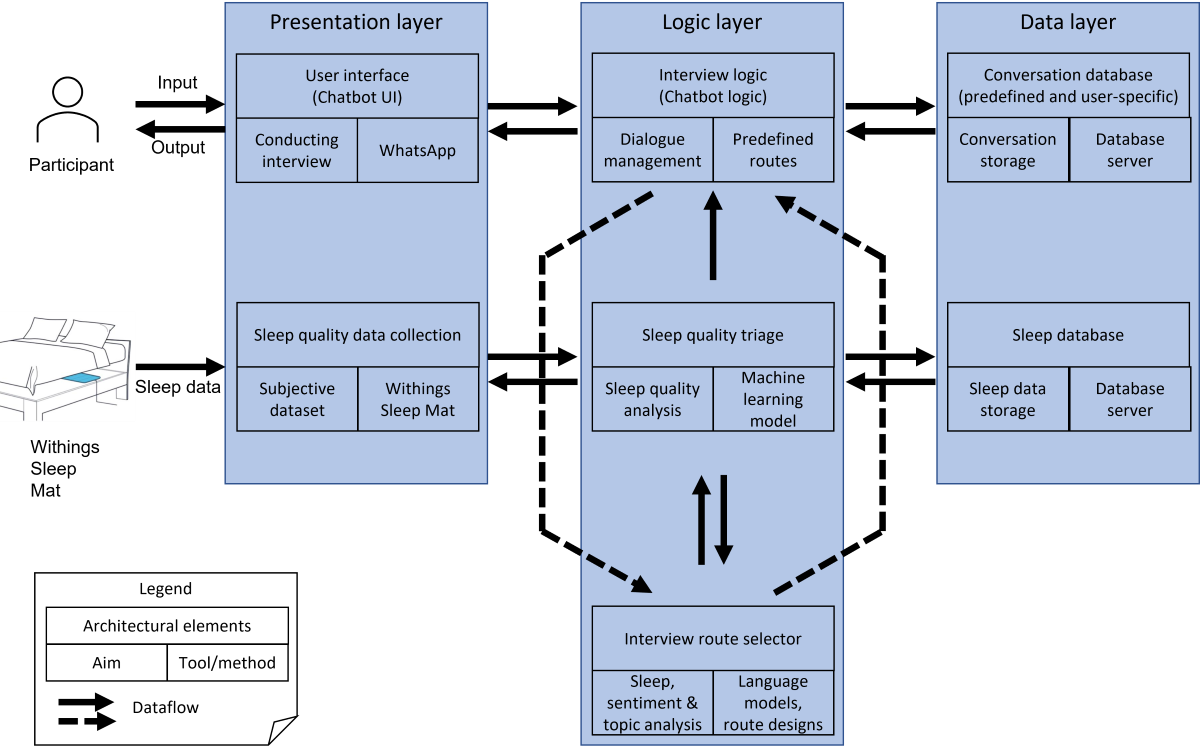
The architecture of the proposed MotivSleep chatbot. UI: user interface.

The chatbot component of our proposed MotivSleep chatbot will automatically start a conversation with the user at least twice a week, on Wednesdays and Saturdays, to prevent data scarcity and ensure we collect data from all participants. In addition to the previously mentioned 2 days, the chatbot will start the conversation when the server detects anomalous sleep patterns from the participant’s data. Other than on these occasions, MotivSleep will not start the conversation to avoid too many repetitive requests for information from participants. Furthermore, participants can trigger the chatbot at any time by initiating the chat (ie, by sending “Hi MotivSleep” to the chatbot). The architecture of MotivSleep is shown in Figure 1 and is based on the Distinguish Business Logic from ML Model pattern [30].

Specifically, MotivSleep consists of 3 layers: a presentation layer, a logic layer, and a data layer, as follows:

1. The presentation layer provides a user interface to conduct interviews where participants interact with MotivSleep delivered through WhatsApp. Backend structures allow MotivSleep to acquire sleep data from the Withings sleep analyzer.
2. The logic layer that provides the chatbot logic to manage the chatbot dialogue based on predefined dialogue routes, a sleep assessments triage module that analyzes participants’ sleep assessments using pretrained machine learning models, and an interview route selector that selects conversation paths based on sentiment analysis and topic modeling using machine learning models.
3. The data layer that provides data storage for participants’ conversation history and sleep data gathered from the Withings sleep analyzer.

### Chatbot Features

The participants will interact with the WhatsApp chatbot and have a Withings sleep analyzer installed to track their sleep. Our system’s backend server is connected to the Withings sleep analyzer database. The sleep monitoring data is accessible through the designed triage mode and the chatbot feature, with which the participants can interact. Specifically, MotivSleep accesses participants’ sleep-tracking data in the morning and triages their objective parameters. The triage will result in three signals, as follows:

1. Red: an anomalous night with significant differences from the usual (ie, gathered 2 weeks before the start of the chatbot engagement) patterns is detected. Specifically, this relates to participants’ bedtime, wake-up time, the number of times they leave the bed during the night, and the duration of snoring time.
2. Yellow: when the model detects ongoing problems in their sleep.
3. Green: when there are no significant differences in sleep patterns or other existing sleep problems.

**Figure 2:**
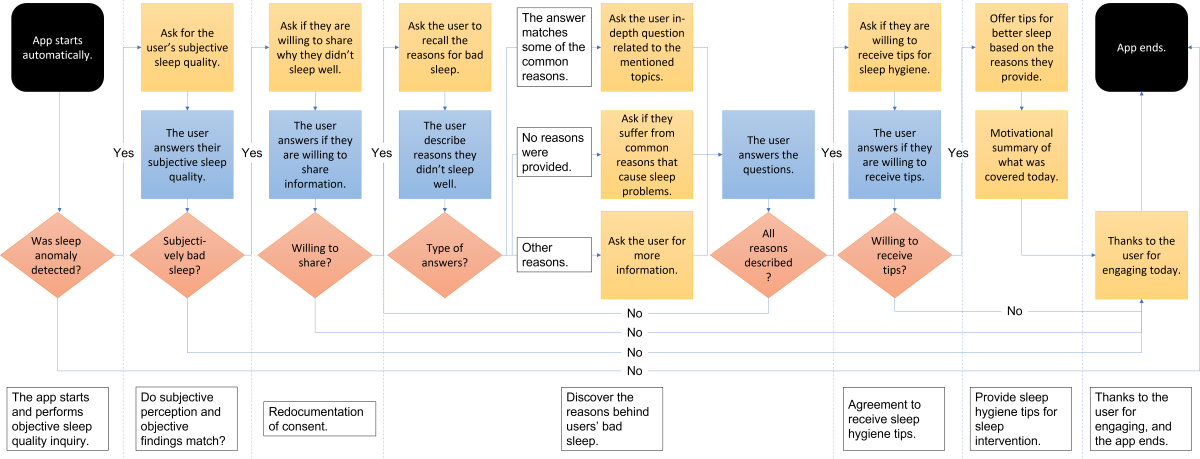
Chatbot dialogue design. Note that the black ovals denote the start and the end of the skill, the yellow rectangles denote the dialogue by the chatbot, and the blue rectangles and the blue diamond denote the participants' inputs. The orange diamonds denote the diverging options.

The MotivSleep chatbot follows a dialogue route with multiple branches, which may end or continue the conversation. Figure 2 shows the dialogue design of our chatbot. Specifically, our chatbot is designed with seven stages, as follows:

1. The objective sleep assessment: sleep quality is highly challenging to assess, with a range of potential models for data fusion from the internet of things devices. In the context of our study, we will initiate analyses of the objective sleep assessment using measures provided by the installed Withings sleep analyzer. Specifically, MotivSleep starts at a predefined time each day and calculates the sleep quality using the sleep tracking device. If the triage result is red or yellow, MotivSleep will continue to the next stage to start the chatbot and engage with the participant; otherwise, MotivSleep will not send the initial text message and will quit automatically. We note that the UK DRI CRT has ongoing work in sleep quality evaluation using the Withings sleep analyzer, which could be incorporated into the study for comparison when available.
2. The subjective sleep assessment: MotivSleep chatbot activates the interview through WhatsApp and asks the participant, “Did you sleep well last night?” This stage helps MotivSleep confirm with the participant if they had a bad night’s sleep. If the participant denies having had a bad sleep, our chatbot will go to stage 7, wishing them a good day and quitting. However, if the participant confirms that they have had a bad night, the chatbot will continue to ask the next question.
3. Redocumentation of consent: the chatbot will briefly remind the participant about the study, ask them for their consent to participate in the current interactions, and provide insights into their poor sleep. If they do not consent, the chatbot will go to stage 7, where we thank them for their engagement, and the chat ends; otherwise, the chatbot will continue to the next stage.
4. The information-seeking stage: in this stage, the chatbot will ask the participant for reasons that might explain why they did not sleep well the previous night. This open question seeks insights into participant’s behaviors before and during their sleep that may have contributed to a bad night’s sleep. Answers to this question are collected and analyzed, and the analysis result will prompt the following three types of further questions:
  - In-depth questions related to predefined specific reasons that cause their poor sleep. These questions will be triggered if we identify predefined reasons from the participant’s answers;
  - Multiple-choice questions consist of potential reasons that might cause poor sleep. These questions will be asked if the participant answers that they do not know why they had a poor sleep;
  - Similar questions to the previously asked question (ie, why did you sleep poorly?) until they confirm there is no more information to add. These questions will be asked if the reasons the participant provided early are not one of the predefined reasons.
5. Agreement to receive personalized recommendations for better sleep: in this stage, the chatbot will ask the participant whether they want to hear personalized recommendations for better sleep. If they disagree, the chatbot will go to stage 7, where we thank them for their engagement, and the chat ends; otherwise, the chatbot will continue to the next stage.
6. Provide personalized recommendations for better sleep: in this stage, we provide specific recommendations to the participant based on the information gathered during stage 4. Furthermore, we provide a motivational summary of what was covered today, including their reasons for poor sleep and recommendations for better sleep.
7. The end of the conversation: in this stage, we thank the participant for the engagement, wish them a good day, and end the conversation. To further motivate the participants, the chatbot also praises the participant if some of the repeated behaviors negatively affecting their sleep were not mentioned in this interaction.

This chatbot aims to perform knowledge transfer, that is, using the information gathered from the fourth stage to provide personalized recommendations for better sleep in the sixth stage, thus transferring knowledge on better sleep to the participants.

### Research Questions

The MotivSleep chatbot used in this feasibility study has been co-designed with patients, carers, and other health professionals. Together, we identified the following research questions:

1. *RQ1* Is our proposed MotivSleep chatbot accessible to participants, and how feasible is it to engage the participants with interviews using MotivSleep? We focus on assessing the feasibility and usability of our proposed MotivSleep, by measuring the number of turns and time spent chatting with the skill per engagement. Moreover, we assess MotivSleep’s accessibility by asking participants to rate their willingness to promote the chatbot using the net promoter score (NPS) [31] question in the exit questionnaire. Finally, we assess the participants’ perception of the chatbot by asking participants to complete the Godspeed Perceived Intelligence subscale (GPIS) [32] and Trust Perception Scale-Human Robot Interactions (TPS) [33] questionnaires.
2. *RQ2* Can our proposed MotivSleep chatbot help improve participants’ subjective sleep assessment? We aim to evaluate the participants’ self-reported sleep assessment to analyze if the proposed MotivSleep helps participants to subjectively sleep better through the completion of the Epworth sleepiness scale (ESS) [34], Insomnia Severity Index (ISI) [35], modified Pittsburgh sleep quality index (PSQI) [36], and the single-item sleep quality scale (SQS) [37], conducted at the beginning, the end, and every 2 weeks throughout the study.
3. *RQ3* Can our proposed MotivSleep chatbot help improve participants’ sleep, measured using the Withings sleep analyzer? We aim to analyze participants’ objective sleep assessments using sleep recordings gathered by Withing sleep analyzer throughout the study.

## Methods

### Overview

Once reviewed by the Imperial College Research Ethics Committee, we will recruit 30 healthy older adults (aged 65-90 years) in 3 cohorts through the UK DRI CRT community outreach group community outreach in West London. Participants will have access to the conversational agent through WhatsApp chat. We estimate that the average conversation will take less than 10 minutes. Each participant will also have access to the Withings sleep analyzer provided by the research group.

A poststudy questionnaire consisting of the GPIS, TPS, and NPS will be completed when the participant finishes the study. We analyze participants’ answers to assess the participants’ opinions on the proposed MotivSleep chatbot’s intelligence, trustfulness, and participants’ satisfaction with MotivSleep, to answer RQ1. Additionally, all participants will complete the ESS, ISI, modified PSQI, and SQS questionnaires before the start, fortnightly during the study, and at the end of the study. We analyze the answers to these questions to investigate RQ2.

The first cohort of 10 participants will use both the Withings sleep analyzer for 6 weeks (the first 2 weeks aims to establish baseline sleep assessments without the usage of MotivSleep) and the WhatsApp chatbot for 4 weeks, with WhatsApp message reminders to encourage WhatsApp chatbot engagement sent out fortnightly (ie, twice for the first cohort of 10 participants). The first batch of participants’ feedback and engagement measures will inform a new iteration of improvements.

The second cohort of 10 participants and the third cohort of 10 participants will use the WhatsApp chatbot for 8 weeks and 12 weeks and the Withings sleep analyzer for 10 weeks and 14 weeks, respectively. Participants will be reminded fortnightly (ie, 4 times for the second cohort participants and 6 times for the third cohort participants) to encourage participation with the WhatsApp chatbot when activated. After each cohort, participants’ technical feedback will be addressed by improving the software.

The feasibility study will have a single experiment condition that includes three types of features:

1. A sleep tracking device (ie, Withings sleep analyzer) and sleep analysis model.
2. A chatbot (ie, MotivSleep) hosted on WhatsApp that conducts sleep interviews. The dialogue is designed to understand the reason behind participants’ sleep problems, provide personalized recommendations for better sleep, and support behavior changes to improve their sleep assessments.
3. WhatsApp messages that encourage interaction with MotivSleep.

We will measure three classes of outcome variables as follows:

1. Assess the feasibility and usability of our proposed chatbot MotivSleep (RQ1)—the number of interactions in each MotivSleep conversation and time spent chatting with MotivSleep, and the poststudy questionnaire consisting of NPS, GPIS, and TPS.
2. Assess the subjectively perceived sleep assessments (before, during, and after the study) (RQ2)—ESS, ISI, PSQI, and SQS questionnaires.
3. Assess the objectively measured sleep assessments (before, during, and after the study) (RQ3)—obtained from the Withings sleep analyzer and analyze using the Withings provided sleep analysis models.

### Recruitment

This study will be conducted in 3 batches. Participants in all 3 batches will be healthy older adults with no neurodegenerative disease. The participants will be recruited through the UK DRI CRT community outreach group in West London. The recruitment process follows the recommendations and requirements laid out in the UK National Institute for Health and Care Research Clinical Research Network policy, based on the inclusion and exclusion criteria. Specifically, Participants should be aged between 65 and 90 years, able to communicate fluently in English, experiencing sleep problems and wanting to improve their sleep, willing to install a Withings sleep analyzer and use the WhatsApp chatbot and have access to high-speed wireless internet connection at home, and able to provide informed consent. The exclusion criteria include the following: (1) people with unstable mental states, including severe depression, severe psychosis, agitation, and anxiety; (2) people with severe sensory impairment; (3) people who have active suicidal thoughts; (4) people who are receiving treatment for terminal illnesses.

Participants will be compensated for their time and inconvenience with £25 (US $31.15) per 2 weeks for participation, that is, for filling out the prestudy informed consent and questionnaires before, during, and after the study and engaging with the chatbot. The questionnaires will be provided on Qualtrics, separately to the chatbot, and sent to the participants as WhatsApp messages. Additionally, our team will teach the participants to complete the questionnaire using their smartphones before the study on an in-person visit when they sign the consent form. The subsequent questionnaires during the study will be sent to their mobile as WhatsApp messages.

A total of 30 individuals will be recruited in 3 cohorts. In total, we aim to include 18% of individuals who self-identify as members of a minority ethnic group to ensure alignment with the UK population [38] and avoid exclusion. Only approved phone numbers (of people who have consented to the study) can interact with the chatbot.

### Data Management

WhatsApp messages are automatically encrypted, so conversations are private and cannot be accessed by Meta (the mother company of WhatsApp).

We will create 2 databases in Microsoft Azure, where both databases are only accessible through designated virtue private network connections, which are available only to the app and the researchers. We will use the Research Data Storage (RDS) managed by the Imperial College London to host the data sets after obtaining them through Azure. RDS is only available to researchers within the project through university-hosted Virtual Private Network connections.

Specifically, we will first pseudoanonymize each user by assigning them an automatically generated user ID and storing this information in a secure Azure database. MotivSleep then automatically stores the transcripts anonymously in a second secure database. Both databases are hosted on Azure before being transferred to RDS. After moving both user information and transcripts to RDS, they will be permanently deleted from the Azure databases. There will be 2 copies of sleep tracking data, that is, Withings will store 1 copy, and the RDS will store another copy. Responses to the questionnaires will be stored in Qualtrics servers.

According to the Imperial College data retention policy, the data will be retained for at least 12 years.

### Analysis Plan

We aim to analyze the following four types of data:

1. The dialogue transcripts: dialogue transcripts can help us understand how much time participants spend talking to the chatbot, what sleep behaviors each participant exhibits, and how often these behaviors appear when troublesome sleep patterns are detected.
2. The answers to the GPIS and TPS questionnaires: these answers help us to understand how participants perceive our proposed chatbot MotivSleep, in terms of usability, feasibility, intelligence, and trustfulness.
3. The answers to the ESS, ISI, modified PSQI, and SQS questionnaires before, during, and after the study help us analyze participants’ subjective views of their sleep assessments and provide insights into their perceived sleep changes over time.
4. The sleep data tracked by the sleep tracking device Withings sleep analyzer: Sleep tracking helps us monitor participants’ objective sleep assessment changes over time, measured by the tracking devices unrelated to participants’ subjective feeling of their sleep.

### Ethical Considerations

The study will be submitted to the Imperial College Research Ethics Committee at Imperial College London, London, United Kingdom. All participants will be provided with an information sheet detailing the aim and rationale for the research; inclusion and exclusion criteria; what will happen if they agree to take part; any risks of taking part in the study; clarification of the process for ensuring anonymity and confidentiality, including any limits to confidentiality; what information will be held about them and who will have access to it; possible benefits; and plans for project report. All participants will then be required to sign the informed consent form before participating in the study. Additionally, all participants’ identifiable data will first be anonymized. The identifiable and anonymized data will be stored in separate secure cloud-based databases (Azure PostgreSQL server) before being transferred to Research Data Store. Finally, each participant will be compensated £25 (US $31.15) per 2 weeks for their participation.

## Results

The planning and development of this study started in August 2022. We have since developed the chatbot MotivSleep. The chatbot’s design is being evaluated by carers, health care professionals, and occupational therapists and will be updated after their evaluation. The first cohort of participation recruitment will start in May 2023, the second cohort will begin in June 2023, and the third cohort will start in August 2023. The anticipated study completion date is December 2023.

In this study, we aim to test three hypotheses as follows:

1. First, we hypothesize that participants will perceive our chatbot as intelligent and trustworthy and are satisfied with our proposed MotivSleep.
2. We also hypothesize that our proposed chatbot MotivSleep can improve participants’ subjective sleep assessment over their usage of the chatbot based on collected using ESS, ISI, PSQI, and SQS questionnaires and their objective sleep quality based on the Withing sleep analyzer.
3. Finally, we hypothesize that participants’ objective sleep assessment will improve, measured by the Withings sleep analyzer.

## Discussion

### Principal Findings

Sleep disturbances are commonly observed among older people, with and without neurodegenerative disorders, and among carers for people with neurodegenerative disorders. This study will provide critical evidence pertaining to the feasibility and acceptability of the proposed chatbot MotivSleep, hosted on WhatsApp, which aims to conduct automatic sleep interviews and provide personalized recommendations for better sleep that may improve their sleep. Specifically, we evaluate the participants’ perceived usability and feasibility of the chatbot, changes in participants’ subjective sleep assessment before, during, and after the study, and changes in participants’ objective sleep assessment measured by the sleep tracking device Withings sleep analyzer.

This paper describes the protocol used in the MotivSleep study. To the best of our knowledge, no other studies have focused on developing an automated chatbot for older people with problematic sleep or conducting automated sleep interviews. The MotivSleep study aims to shed light on the feasibility of using a chatbot to conduct interviews for a prolonged period of time. Furthermore, the intervention and protocol were designed to be more accessible and simpler than frequent in-person assessment or intervention sessions. This allows future deployment to older adults with mobility issues or within hard-to-reach communities.

### Limitations

Our study requires participants to use 2 devices (a Withings sleep analyzer and a smartphone to access WhatsApp) and complete web-based questionnaires. As such, older people unfamiliar with or unwilling to use these devices might self-exclude. However, successful findings from this study could be leveraged to revise further to allow for more accessible options on the technological design for a broader audience and users. Additionally, excluding participants with diagnosed life-threatening illnesses and severe mental health problems is a limitation. However, for this preliminary study, such exclusion is warranted to ensure that participants are medically stable and not reporting severe clinical symptoms that would require a higher level of care, which a digital chatbot cannot help treat.

Furthermore, we acknowledge the small number of participants in this feasibility study. Thus, we do not expect to find significant differences with respect to gender or age. However, the proposed sample size aligns with previous research on the number of participants needed to assess usability and feasibility.

### Conclusions

To conclude, the MotivSleep study is innovative in using an automated chatbot to conduct sleep interviews for older people who wish to improve their sleep. Evidence of its feasibility and acceptability will facilitate a larger trial. Participants perceived changes and sleep tracking devices’ recorded changes will provide early evidence of its effectiveness. Moreover, the MotivSleep chatbot has the potential to be deployed by a large number of people due to its automated nature. This can offer older people access to supportive care that is more easily accessible and more affordable than face-to-face therapy with sleep experts.

## Abbreviations

CRT: Care Research and Technology Center
ESS: Epworth sleepiness scale
GPIS: Godspeed Perceived Intelligence subscale
ISI: Insomnia Severity Index
mHealth: mobile health
NPS: Net Promoter Score
PSQI: Pittsburgh sleep quality index
RDS: Research Data Storage
SQS: single-item sleep quality scale
TPS: Trust Perception Scale
UK DRI: UK Dementia Research Institute

## Data Availability

The sleep anomaly detection model is developed based on sleep records from more than 3.6 million nightly summaries from 13,671 individuals from the general population who use the Withings sleep analyzer, which is unavailable to the community due to the Withings Company’s policy. However, based on the ethics approval, dialogue data will be made available upon request to support scientific transparency.
